# Effects of Oil Supplements on Growth Performance, Eating Behavior, Ruminal Fermentation, and Ruminal Morphology in Lambs during Transition from a Low- to a High-Grain Diet

**DOI:** 10.3390/ani12192566

**Published:** 2022-09-26

**Authors:** Leili Bahramkhani-Zaringoli, Hamidreza Mirzaei-Alamouti, Jörg R. Aschenbach, Mina Vazirigohar, Amlan Kumar Patra, Iraj Jafari-Anarkooli, Mahdi Ganjkhanlou, Daryoush Alipour, Morteza Mansouryar

**Affiliations:** 1 Department of Animal Science, Faculty of Agriculture, University of Zanjan, Zanjan 45371-38791, Iran; 2 Institute of Veterinary Physiology, Freie Universität Berlin, Oertzenweg 19b, 14163 Berlin, Germany; 3 Zist Dam Group, University of Zanjan Incubator Center, Zanjan 45371-38791, Iran; 4 Department of Animal Nutrition, West Bengal University of Animal and Fishery Sciences, Belgachia, Kolkata 700037, India; 5 Department of Anatomical Sciences, School of Medicine, Zanjan University of Medical Sciences (ZUMS), Zanjan 45139-56111, Iran; 6 Department of Animal Science, Faculty of Agriculture, University of Tehran, Karaj 77871-31587, Iran; 7 Department of Animal Science, Faculty of Agriculture, Boalisina University, Hamedan 65178-33131, Iran

**Keywords:** rumen, palm oil, soybean oil, papilla, lamb

## Abstract

**Simple Summary:**

Feed efficiency is generally associated with a considerable consumption of grains by ruminants. However, high-grain feeding may induce digestive problems in ruminant animals. The gradual adaptation to high-grain diets can be considered an effective strategy to reduce these issues. Free-oil supplementation in the adaptation diets of lambs is not common, but may smooth the transition from a high-forage to a high-grain diet, which was evaluated in this study. Lambs received diets without free-oil or containing palm or soybean oil (80 g/day) during and after the adaptation period. In lambs fed free-oil, particularly soybean oil, dry matter intake, daily weight gain, and ruminal pH increased, while concentrations of branched chain fatty acids in the rumen decreased, and the feed efficiency, as well as development of ruminal epithelia, improved compared with those fed the control diet.

**Abstract:**

The objectives of this study were to investigate the effect of a maximum recommended oil supplementation on growth performance, eating behavior, ruminal fermentation, and ruminal morphological characteristics in growing lambs during transition from a low- to a high-grain diet. A total of 21 Afshari male lambs with an initial body weight (BW) of 41.4 ± 9.1 kg (mean ± SD) and at 5–6 months of age were randomly assigned to one of three dietary treatments (*n* = 7 per group), including (1) a grain-based diet with no fat supplement (CON), (2) CON plus 80 g/d of prilled palm oil (PALM), and (3) CON plus 80 g/d soybean oil (SOY); oils were equivalent to 50 g/kg of dry matter based on initial dry matter intake (DMI). All lambs were adapted to the high-grain diet for 21 d. In the adaptation period, lambs were gradually transferred to a dietary forage-to-concentrate ratio of 20:80 by replacing 100 g/kg of the preceding diet every 3 d. Thereafter, lambs were fed experimental diets for another 22 days. Fat-supplemented lambs had greater DMI, body weight (BW), and average daily gain (ADG), with a lower feed to gain ratio (*p* < 0.05), compared to CON lambs. The highest differences of DMI between fat-supplemented and CON-lambs were observed in week 3 of the adaptation period (*p* = 0.010). PALM- or SOY-supplementation lowered DM and NDF digestibility compared with CON (*p* < 0.05), and SOY caused the lowest organic matter (OM) digestibility compared with CON and PALM lambs (62.0 vs. 67.6 and 66.9; *p* < 0.05). Ruminal pH was higher for PALM and SOY compared with CON (*p* = 0.018). Lambs in SOY tended to have the highest ammonia-N concentrations (*p* = 0.075), together with a trend for higher concentrations of propionic acid, at the expense of acetic acid in ruminal fluid, on the last day of the adaptation period (diet × time, *p* = 0.079). Fat-supplemented lambs had lower isovaleric and valeric acid concentrations compared with CON on d 40 (diet × time, *p* < 0.05). PALM and SOY-fed lambs had a longer eating time (min/d and min/kg of DMI), chewing activity (min/d), meal frequency (n), and duration of eating the first and second meals after morning feeding (*p* < 0.05), and the largest meal size (*p* < 0.001). Fat supplemented lambs had greater ruminal papillary length (*p* < 0.05) and width (*p* < 0.01), and thicker submucosal, epithelial, and muscle layers, compared with the CON (*p* < 0.01). Blood metabolites were not influenced by dietary treatments (*p* > 0.05). The results from this study suggest that fat supplementation to high-grain diets may improve the development of ruminal epithelia and modify ruminal fermentation via optimized eating behavior or the direct effect of oils on the ruminal environment, resulting in better growth performance in growing lambs.

## 1. Introduction

Maximizing animal performance, without harming animal health and welfare, is one of the most challenging aims in livestock production. This goal is even more critical in ruminant production, since the rumen as a complex organ has its specific needs for adaptation and adjustment. Adequate diet provision, with optimized dietary changes, is amongst the most critical challenges during the different physiological stages. For example, in growing feedlot ruminants, the transition from a forage- to a concentrate-based diet can be a troublesome: If the dietary transition does not occur properly, this can lead to inadequate or delayed adaptation of ruminal microbes and physiological functions of the ruminal epithelium (permeability, proliferation, activity of transport proteins, and metabolic activity). In severe cases, ruminal or systematic inflammation can occur [1,2]. High grain consumption increases short chain fatty acid (SCFA) concentrations in the rumen, reduces ruminal pH, decreases the absorption capacity of the ruminal epithelium, and impairs tight junction function in the rumen [2,3]. These conditions can lead to incomplete formation of digestive layers in the rumen, reduced chewing activity, decreased salivation, declined ruminal wall movements, and decreased ruminal mixing of digesta [4]. A decreased ruminal pH, concurrent with high SCFA concentrations, creates an acidotic condition in the rumen, which impacts animal performance negatively [5,6]. For a safe transition, the classic recommendation is to increase the plane of fiber in the diets [1]; however, this approach can limit animal energy intake, which is the most limiting factor for efficient production in feedlot ruminants [7]. During the adaptation period, energy intake is increased by replacing concentrate for forage; however, the daily energy intake is often low at the beginning of such dietary transitions. In addition, increasing the grain portion as an energy source may impair ruminal development, as previously mentioned [2,3]. Therefore, implementing nutritional interventions in the transition period to high-grain diets is important for improving ruminal function and animal performance.

Some studies suggested that supplementation of fat can be a suitable alternative; as fats may increase feedlot cattle energy intake [8], change eating behaviors in dairy cows [9], microbial communities, and expression of genes in the ruminal epithelium of growing lambs [10], and may prevent overproduction of SCFA and lowering of ruminal pH in growing lambs [10,11]. However, studies in which fat supplements (FS) were added to counteract the effect of highly fermented grains in the transition diets of growing lambs are scarce, and no such study investigated ruminal morphological structural changes. Recently, Mirzaei-Alamouti et al. [10] supplemented a mixture of fish and sunflower oils at the maximum recommended amount (50 g/kg of DM) to a high-concentrate finishing diet and found an improvement in rumen fermentation and a stabilized ruminal pH in growing lambs (8 months age). An increased ruminal pH was accompanied by a low acetate to propionate ratio. Another study reported that inclusion of yellow grease into the basal diets counteracted the negative effects of low-energy intake and increased the feed efficiency of newly received feedlot calves [12]. However, Toral et al. [13] reported that inclusion of a mixture of fish and sunflower oils (30 g/kg of DM) to a high concentrate diet had no effect on ruminal pH and SCFA concentrations in ewes. In addition, during adaptation to high-grain diets, feeding an adequate amount of a fat supplement (80 g/d for each lamb or 50 g/kg of DM), concurrent with decreasing forage-to-concentrate ratios, may increase the daily energy intake of lambs, especially in the early days of this dietary change, when the concentrate consumption is low. Moreover, the type of fat supplement, determined by the degree of saturation and carbon length, greatly affects the feed digestibility, ruminal fermentation, and microbial communities in the rumen [14,15]. 

In the present study, it was therefore hypothesized that feeding a constantly high amount (80 g/d) of plant oil, along with decreasing the forage-to-concentrate ratios in diets, would not only counteract the negative effect of a high-concentrate diet on rumen fermentation (indexed by rumen fermentation characteristics, eating behavior, and rumen epithelium morphology), but might have additional benefits for lamb performance and efficiency. Furthermore, fats with a high concentration of saturated (e.g., palm oil) versus unsaturated (e.g., soybean oil) fatty acids may produce differential responses for the above attributes. Thus, the aim of this study was to investigate the effect of the daily feeding of 80 g/d palm or soybean oils on growth performance, ruminal fermentation characteristics, nutrient digestibility, eating behavior, blood metabolites, and ruminal papillae morphology in growing lambs transitioned from a low- to high-grain diet over a practically relevant period of 21 d. 

## 2. Materials and Methods

### 2.1. Animals and Feeding Management

The experimental and management protocols of this study were approved by the animal care and welfare committee (ID 1353) at the University of Zanjan, Iran. A total of 21 Afshari male lambs with an initial body weight (BW) of 41.4 ± 9.1 kg (mean ± SD) and approximately 5–6 months of age were assigned to one three dietary treatments (n = 7 lambs per treatment) in a completely randomized design: (1) control diet (grain-based diet without fat supplement, CON), (2) control diet plus 80 g/d of prilled palm oil (PALM; Energizer RP 10, Malaysia), and (3) control diet plus 80 g/d of soybean oil (SOY). The fat supplements were equivalent to 50 g/kg of dry matter, based on the initial dry matter intake (DMI). Initial DMI for four days before the adaptation period was used to determine the daily amount of oil supplement for lambs during the experimental period. Oils replaced other dietary ingredients on a proportionate basis. The ingredients of the basal diet are shown in Table 1 (154 g/kg of crude protein, CP; 11.6 MJ/kg of metabolizable energy, ME). Concentration of ME increased with the addition of the oil (12.5 MJ/kg) and other nutrients decreased. CNCPS-S software was used for diet formulation (version 1.0.21; Cornell University, Ithaka, NY), to achieve a high growth rate. Lambs were kept individually in separate pens (171 × 83 cm) with a concrete floor and equipped with fresh water and feed bunks. All lambs had an initial period of 4 d, used for the recording of initial parameters as covariates. Thereafter, lambs had an adaptation period of 21 d to adapt to the high grain-diet and received a constant amount of 80 g/lamb per day oil supplement, and then the lambs continued to be fed the high concentrate diet plus oil supplement until d 43. During the adaptation period, lambs were gradually transferred from a dietary forage-to-concentrate ratio of 80:20 to 20:80. The preceding diet (containing: alfalfa hay, 800 g/kg of DM, and whole barley grain, 200 g/kg of DM) was gradually replaced by an increasing fraction (plus 100 g/kg per 3 d) of the concentrate diet. Forage and concentrate portions for each lamb were hand-mixed daily and offered to the lambs once a day at 09:00 am. The oil supplements (80 g for each lamb/d) were mixed with the concentrate portion in the bunk daily. The diets offered and orts of each lamb before morning feeding were sampled to determine the dry matter and nutrient contents. All lambs had ad libitum access to feed and fresh water. Lambs were visually monitored every day, and their health status, such as laminitis and diarrhea, was recorded. After slaughter, the liver was evaluated for the presence of abscesses. 

### 2.2. Animal Performance

The diets offered and refusals were weighed daily before the morning feeding, and the DMI for each animal was calculated. Individual BW was recorded weekly after 16 h of water and feed deprivation, to determine the average daily body weight gain (ADG) and feed to gain ratio (F:G). 

At the end of the feeding period, all lambs were slaughtered at the slaughterhouse of the University Farm Animal Research and Teaching Station. The animal weight before slaughter was recorded as slaughter body weight (SBW). Immediately after slaughter, all the thoracic and abdominal organs were removed and the animals were skinned. The total non-carcass parts were removed and the hot carcass weight (HCW) was recorded, and the hot dressing percentage was obtained using the ratio of (HCW/SBW) × 100. 

### 2.3. Dry Matter and Nutrient Digestibility

An internal marker method was used to determine total tract apparent digestibility of nutrients. Acid insoluble ash (AIA) as an internal marker was measured in feed and fecal samples, to determine the coefficient of apparent DM and nutrient digestibility [16]. Fecal samples were taken directly from the rectum every 6 h over a period of 48 h (i.e., 4 times/day) on d 23 and 24. After oven-drying at 55 °C for 24 h, feed and fecal samples were mixed and ground to pass through a 1-mm screen using a Wiley mill (Arthur H. Thomas Co., 153 Philadelphia, PA, USA). Subsequent nutrient analysis utilized the standard methods of AOAC [17]: method 934.01 for DM, method 976.05 for CP, method 942.05 for ash, method 920.29 for ether extract (EE), and the method of Van Soest et al. for heat-stable α-amylase NDF [18]. 

### 2.4. Ruminal Fluid and Blood Sampling and Analysis

To evaluate the ruminal fluid content, sampling of all animals was performed by applying vacuum pressure to an esophageal tube fitted with a suction strainer on d 0, 13, 20, and 40 of the experiment. During the 21-d dietary adaptation period, 10 percent concentrate was increased every 3 d, and the 10 percent forage fraction was consequently reduced. Therefore, on the days of ruminal fluid sampling, the F:C ratios were 80:20 on day 0, 40:60 on d 13, and 20:80 on d 20 and 40. To minimize saliva contamination, approximately 200 mL of initial ruminal fluid was discarded before sample collection. The ruminal fluid content was filtered through a four-layer cheese cloth and immediately subjected to pH measurement (ABB Kent Taylor, Kent EIL, UK). An 8-mL aliquot of strained sample was mixed with 2 mL of 25% (wt/vol) metaphosphoric acid and frozen at −20 °C, until being analyzed for concentrations of short-chain fatty acids (SCFA) using a gas chromatograph (GC). A second 8-mL aliquot of strained ruminal fluid samples for the measurement of ammonia nitrogen was also obtained, mixed with 2 mL of 25% (vol/vol) sulfuric acid (molar mass 98.07), and kept at −20 °C. 

Rumen fluid sampling was performed at 3 to 4 h after feeding, to monitor the maximum concentration of SCFA. To analyze the concentrations of SCFA, the acidified ruminal fluid samples were thawed, shaken, and allowed to settle for 15 min at room temperature. A 5-mL aliquot of ruminal fluid supernatant and 1 mL of meta-phosphoric acid-internal standard (2-ethyl butyric acid, Sigma-Aldrich, Darmstadt, Germany) solution were mixed and transferred into a 15-mL glass test tube. The tube was centrifuged at 12,000× *g* and 4 °C for 15 min. The prepared samples were transferred into an Eppendorf tube, and a 1-μL aliquot of the upper layer was injected into a GC (Varian 3400, Varian Inc., Walnut Creek, CA), equipped with an injector at 170 °C, a flame-ionization detector at 175 °C, and a packed column (6’ × 2 mm ID glass containing 1–1965 10% SP-1200/1% H_3_PO_4_ on 80/100 Chromosorb W, Varian Inc., Walnut Creek, CA). The temperature of the GC oven was isothermal and maintained at 140 °C. Gas flow rates were 40 mL/min for nitrogen and 300 mL/min for compressed air [19]. To determine the concentration of ammonia-N in the ruminal fluid samples, the procedure of Broderick and Kang [20] was used with a minor modification, where manganese sulfate was used as a catalyst instead of nitrous oxide. 

To assess selected key metabolites responsive to the adequacy of the nutrient intake, blood samples were taken 3 h after feeding on d 0, 15, 25, and 40 of the experiment in heparinized vacuum tubes from the jugular vein. To collect plasma, samples were immediately centrifuged (3000× *g* at 4 °C for 20 min) and transferred to 2-mL microtubes and stored at −20 °C until analysis. Colorimetric methods were applied to determine plasma concentrations of glucose, cholesterol, albumin, and total protein using commercial kits (Pars Azmun Co., Tehran, Iran), by following the manufacturer’s protocols (PerkinElmer, Colemen Instruments Division, Oak Brook, IL, USA).

### 2.5. Eating Behavior

All eating behaviors were monitored visually over a 24-h period on d 22 by three trained observers. Behaviors, including eating, ruminating, and chewing, were recorded with 5-min interval observations, and each activity was assumed to persist for the entire 5-min interval. Eating, ruminating, and chewing times; eating and ruminating lengths; meal frequency; ruminating bouts; largest meal; and the length of the first and second meal after morning feeding were reported.

### 2.6. Morphometry of Ruminal Papillae

Immediately after slaughter and removal of the abdominal organs, tissue samples (2 to 3 cm^2^) were collected from the ventral sac of the rumen and fixed in 10% formalin. Formalin-fixed samples of tissues were dehydrated in a series of ethanol solutions from 50 to 100% and cleared with xylene. Samples were embedded in paraffin, sectioned with an automatic microtome at 5 µm thicknesses, and stained with hematoxylin-eosin. Histomorphometric analyses were performed by experienced observers blinded to the treatment groups. Morphology was analyzed under a light microscope (Olympus BX-51; Olympus Optical Co., Ltd., Tokyo, Japan) at 40×, 100×, and 400× magnifications. Only paraffin sections with the best transversal orientation of the epithelium were used to evaluate the morphological characteristics. Digital photos of stained sections were taken using an Olympus BX-51 camera (DP 11) and measurements were made using the image analysis computer software Cell (Olympus Optical Co., Tokyo, Japan). The following morphometric values of the ruminal samples were measured: papilla length, papilla width, thickness of the submucosal layer, thickness of the epithelium, and thickness of the muscle layer. Papilla length was defined as the distance from the tip to the base of the papilla, papilla width was defined as the average width of the base, middle, and tip of the papilla. 

### 2.7. Statistical Analysis

Data were tested for normality using PROC UNIVARIATE of SAS software [21]. The MIXED procedure of SAS was used to analyze single time point data, such as eating behavior, digestibility, morphometry, and carcass weight, as well repeatedly measured data, such as DMI, BW, ADG, FCR, blood metabolites, and fermentation characteristics [21]. The statistical model for single time point data was Y_ij_ = µ + Diet_i_+ Lamb_j_(Diet_i_) + e_i_j; where Y_ij_ is the dependent variable, µ is the overall mean, Diet_i_ is the fixed effect of dietary treatment i, Lamb_j_(Diet_i_) is the random effect of lambs nested in the dietary treatment, and e_ij_ is the residual error. Sampling time and sampling time × treatment interaction were included in the model for repeated measurement data, using time as a repeated measure and animal within treatment as the subject. The latter was intended to identify differences in the two experimental phases (adaptation period and post-adaptation period). For each analyzed parameter, a lamb nested within the treatment was subjected to three covariance structures: compound symmetry, autoregressive order one, and unstructured covariance. A variance-covariance structure was chosen based on the best Akaike information criterion. The least square mean (LSM) data are reported and differences among the treatments were compared using a Tukey test. Initial values were added as a covariate to the model, to avoid bias from inter-individual variance. Covariates with a significance probability >0.1 were removed stepwise from the model, one at a time, starting with the least significant in a backwards manner. Contrasts were used to identify differences between CON diet versus both FS diets. Differences between treatments were considered significant and tending to be significant at *p* < 0.05 and 0.05 ≤ *p* < 0.10, respectively. Unless otherwise stated, data are presented as LSM ± standard error of mean (SEM).

## 3. Results

### 3.1. Feed Intake, BW, ADG, F:G Ratio, Carcass Weight, and Lamb Health

The fat supplemented diet increased DMI compared to the CON diet (*p* = 0.028), and intake was influenced by time and the interaction between diet × time (*p* < 0.05; Table 2), with lower DMI in CON compared to SOY or PALM lambs from day 15 to 21 of the adaptation period. Expectedly, the effect of time was significant for BW (*p* < 0.001), since the BW of all lambs increased with progressive days; however, the SOY- and PALM-fed lambs had higher BW (3 kg) compared with CON-fed lambs over the whole period (*p* = 0.006) and differences were significant from d 21 to 46 (*p* < 0.05), as supported by the diet × time interaction (*p* = 0.008). The ADG followed the BW changes, as lambs fed fat supplement had greater ADG compared to the CON lambs (*p* < 0.001). Fat-supplemented diets improved F:G ratio compared with CON diet (*p* < 0.001) over the whole experimental period. Carcass weight was greater in fat-supplemented lambs compared with CON lambs (*p* = 0.005), with no differences in dressing percentages. There was one case each of liver abscess in the CON and PALM groups. Two cases of sever laminitis were recorded in the CON group. Three cases of severe diarrhea were observed in each of the CON and PALM groups after transition to the high-grain diet. 

### 3.2. Digestibility

Apparent digestibility of DM, OM, and NDF was affected by treatments, as PALM- or SOY-supplemented lambs had lower DM and NDF digestibility compared with lambs from the CON-fed group (*p* < 0.05; Table 3). Regarding OM digestibility, however, SOY lambs had the lowest digestibility compared with the two other groups (*p* < 0.05). Digestibility of EE and CP was not influenced by the dietary treatments (*p* > 0.05; Table 3).

### 3.3. Ruminal Fermentation Characteristics

Ruminal pH was lower for CON lambs compared to the other groups (*p* = 0.018; Table 4). Lambs fed the SOY diet showed a trend for diet × time interaction (*p* < 0.1), pointing to higher concentrations of propionic acid at the expense of acetic acid at d 20. Ammonia-N, total SCFA and butyric acid were not impacted by diet or the interaction of diet × time over the whole experimental period (Table 4). Ruminal concentrations of isovaleric and valeric acids were affected by the diet × time interaction (*p* < 0.05), with CON showing higher concentrations than PALM and SOY at day 40.

### 3.4. Blood Metabolites

Diet or the interaction of diet × time had no effect on the concentrations of the tested blood metabolites (Table 5). However, time had a significant effect on glucose, cholesterol, and albumin, as the concentrations of these metabolites were lower on d 40 of experiment than at earlier sampling time points (*p* < 0.001; time data not shown). 

### 3.5. Eating Behavior

After switching to the grain-based diets, eating behavior was assessed at day 22. The addition of PALM and SOY increased eating time (min/d and min/kg of DMI), meal frequency, meal length, duration of eating the first meal after morning feeding, and chewing time (*p* < 0.05; Table 6). The second meals after morning feeding were longest in SOY lambs, whereas PALM lambs had higher ruminating times compared with the other two groups (*p* < 0.05).

### 3.6. Tissue Morphology

The comparison of ruminal papillae morphology between both fat-supplemented groups vs. CON showed that fat supplementation increased papillary length, papillary width, and the thickness of the epithelial, submucosal, and muscular layers (*p* < 0.05; Table 7). Comparison of individual groups for the factor diet identified that SOY lambs had the thickest epithelium and the widest papillae compared with the other groups (*p* < 0.01; Table 7). 

## 4. Discussion

Considering the change in DMI and ADG with the progressing weeks and an increase in the BW of fat supplemented lambs compared with non-supplemented lambs from week 3, we concluded that PALM- and SOY-fed animals had a higher energy and nutrient intake, with improved feed efficiency. There was no difference between the two fat sources, indicating that the different FA profile and the different physical form (solid/prilled vs. liquid) of PALM vs. SOY had no influence on these zootechnical traits. Allen [22] stated that a negative impact of FS on DMI could be due to the decreased ruminal digestibility of OM, causing ruminal fill, an increase in propionate concentration, and oxidation of fatty acids in the liver or greater fatty acid flow into the intestine, and thereby an increased cholecystokinin secretion. However, since fat-supplemented lambs had a lower DM and NDF digestibility compared with the CON group, the increased DMI in this study was more likely related to an increased palatability of the diets supplemented with oils and an improved stability of the ruminal environment (higher ruminal pH), which resulted in a higher daily gain and feed efficiency. Fatty acids may exert antimicrobial effects in the rumen, with more potent effects for unsaturated fatty acids [14,23]. This may result in a decrease in the acetate to propionate ratio, accompanied by reduced digestion of OM, primarily the fibrous fraction [23,24]. This was consistent with our observations that fat supplementation reduced the apparent digestibility of DM, OM, and NDF, with a more pronounced effect from the SOY vs. PALM diet. Decreased nutrient digestibility with the fat supplement diets could also be associated with an increased digesta passage rate from the rumen, given the increased DMI. It is important to note, however, that despite a relative decrease in nutrient digestibility by FS, the absolute amount of digested nutrients did not decrease, but rather increased, which is consistent and explanatory for the observed increase of ADG.

Consistent with our hypothesis, FS had no negative influence on the ruminal fermentation characteristics, and even increased the ruminal pH. Similarly, Mirzaei-Alamouti et al. [10] reported that supplementing a mixture of fish and sunflower oils to finishing lambs increased the ruminal pH after 20 and 30 d of oil introduction. An increase in ruminal pH in their study was concurrent with a linear reduction in concentrations of total SCFA, acetic, iso-valeric, and valeric acids. In the present study, we did not observe a difference in total SCFA concentrations between FS and CON; however, similar to Mirzaei-Alamouti et al. [10], an increase in ruminal pH was paralleled with a remarkable reduction in valeric and iso-valeric acid concentrations. The latter indicates a comparable mode of changes in ruminal fermentation characteristics by FS in high-grain diets. In previous studies [25,26], the proportions of isovalerate and valerate were negatively correlated with ruminal pH. Therefore, the greater proportions of valerate and isovalerate could indicate the susceptibility to subacute ruminal acidosis (SARA) of lambs fed the CON diet. However, there have been large variations in the susceptibility to SARA among lambs on the same diet [27,28]. Occurrence of SARA depends to a large degree on SCFA production and absorption rates [3,5,27]. SCFA are partly products of lactate metabolism by *Megasphaera elsdenii* in the rumen of ruminants receiving high-grain diets [29]. In a recent study, it was shown that the number of *Megasphaera elsdenii* and *Butyrivibrio fibrisolvens* increased in response to fish and sunflower oil supplementation [10], which may suggest that the lower proportions of valeric and isovaleric acids in the FS lambs of the current study may have resulted from selective toxic effects of unsaturated fatty acids and saturated fatty acids on specific bacterial populations, with less toxicity on lactate producing bacteria [30,31]. Additionally, this may have been related to a decreased digestibility of OM, specifically its NDF fraction. Interestingly, propionate, as a major product of the ruminal lactate metabolism [29], did not increase in the CON group and was decreased in the PALM group compared with the SOY. The latter may be related to a previous finding that palmitic acid is toxic to *Prevotella ruminicola* (a major propionate producer) when added to purified bacterial cultures [31]. 

The differences in responses to FS among studies could be due to differences in basal diet composition and the type (fatty acid composition), amount, and duration of FS. In the present study, SOY-fed lambs showed a higher propionic and lower acetic acid concentrations after d 20 of oil introduction. However, this fermentation pattern was not found in PALM-fed lambs. This finding suggests a shift in ruminal fermentation pattern to increased propionate at the expense of acetate concentrations when diets with PUFA-rich oils are fed [10,30,32]. Moreover, duration of FS and forage-to-concentrate ratios seem to be critical for the effect of added fat on ruminal fermentation characteristics. In this regard, and in agreement with Mirzaei-Alamouti et al. [10], we did not find any effect of FS on ruminal fermentation characteristics before d 20 of the adaptation period in which forage was partly and gradually replaced by concentrate. During the adaptation period, lambs consumed a constant amount of FS daily, at an (almost) equal forage-to-concentrate ratio. This might explain why Toral et al. [13] could not detect any effect on ruminal fermentation when adding a mixture of fish and sunflower oils into a high-grain diet for only 10 d in ewes. Thus, dietary, ruminal, and physiological adjustments to fat introduction might be necessary to observe the effects of FS on ruminal fermentation.

The impact of FS on ruminal fermentation pattern, however, is not merely a direct effect of fat on ruminal microbes or ruminal fermentation [10]. In the current experiment, we demonstrated that changes of ruminal fermentation, especially ruminal pH, partly reflected eating behaviors of fat-supplemented lambs. Considering that FS increased DMI, lambs fed SOY or PALM diets spent more time eating and distributed their meal between more eating bouts per day. These findings suggest that the fat-supplemented lambs might have experienced less abrupt changes to their diurnal pH. It has been reported that diets containing PUFA-rich oils can create a hypophagic impact with smaller meal size [9,33]. Intake of smaller and more frequently distributed meals during the day is favorable for ruminal health [1]. In our study, eating behaviors were also affected by the type of supplemented fat. PALM-fed lambs spent more time ruminating (min/d) compared to SOY and CON lambs. PALM- and SOY-fed lambs spent more time chewing compared with the CON. The latter could explain the greater DM intake as rumination time, as the min/kg DM intake was similar among the treatments. In a recent study [34], total ruminating time (min/d) was not affected by different fat sources, but ruminating time as g of DMI/h or g of NDFI/h was found to be greater for calcium salt of fatty acids compared with soybean oil, whole soybean, and corn germ.

Another interesting finding of this study was the morphological impact of FS on ruminal papillae. Morphological parameters, including papillary length; papillary width; and thickness of the submucosal, epithelial, and muscle layers were higher in fat-supplemented lambs compared with the non-supplemented group, with a more pronounced effect of the SOY diet. These changes were further associated with an improved feed efficiency in lambs fed the FS diets. Studies in cattle [35] and sheep [27] showed that high-grain diets can improve ruminal development and function; however, the feeding of high-grain diets may disrupt the function and development of papillae and epithelial cell layers [36] if it leads to SARA that is accompanied by inflammation and accumulation of lipopolysaccharides in the rumen and intestine [37]. The accumulation of LPS in the digesta may damage the integrity of the ruminal and intestinal epithelial barrier, increase epithelial permeability, and thereby affect the ruminal and intestinal structure and microbiota [2,38,39], decrease DMI [40], and increase liver abscesses [41]. These events are associated with high energy and nutrient requirements, which diverts nutrients away from production. 

There is no knowledge on how fat supplementation in transition diets can change ruminal histomorphological parameters and how differences in the ruminal development can influence feed efficiency. In a recent review, Na and Guan [42] summarized the findings on the structure of the ruminal epithelium, epimural microbiota, and epithelial host–microbe interactions, together with their functions and how these are associated with feed efficiency. Unfortunately, the authors did not address the caloric and non-caloric effects of dietary fatty acids. Lam et al. [43] showed that efficient cattle had thicker ruminal epithelial tissues compared with inefficient cattle. Apart from individual variability, ruminal epithelial thickness also depends on diet composition and fermentation products, especially butyrate [44,45,46]. Butyrate concentration was not different among diets in the present study, in line with our previous study [10]; however, all major SCFA likely affect ruminal papillae development [44]. In spite of the lack of differences among diets for the total SCFAs concentration in the rumen, FS diets increased the DMI and ruminal pH, which could be translated to a higher quantity of absorbed SCFA, higher energy supply, and higher feed efficiency. Ruminal SCFA concentration is the result of the production, absorption, washout, and interconversion of SCFA [47]. Lam et al. [43] reported that cattle with a high feed efficiency showed no difference in ruminal SCFA concentration, while they had a thicker ruminal epithelial wall than cattle with a low feed efficiency. Changes in fatty acid composition and enhanced SCFA transport of the ruminal epithelium were reported in Holstein steers fed saturated and unsaturated fat sources compared with non-supplemented steers [48]. There was little evidence for lipid absorption through the ruminal epithelium during a dietary transition from high forage to high grain in beef cattle [49] and lambs [50], based on the expression of genes involved in lipid transport and metabolism. In our previous study [10], oil supplementation of a finishing diet downregulated LPS binding protein and IGFBP-3 in the ruminal epithelia of growing lambs, together with a stimulating effect of dietary oil on epithelial growth. Together, these findings may indicate a causative relationship between the FS, altered fermentation pattern, increased epithelial/mucosal thickness, and improved feed efficiency in the FS lambs of the present study, especially those on the SOY supplement. 

## 5. Conclusions

A high amount of supplemental fat (80 g/d) in the diets of growing lambs experiencing a transition from a high-forage to a high grain-diet resulted in beneficial impacts on ruminal health and development, as well as animal growth performance. We conclude that the positive effects on growth performance were to a great extent the result of an enhanced eating drive, leading to increased DM intake that overcompensated for the concurrently reduced DM and NDF digestibility. The effects of FS on the fermentation characteristics included an increase in ruminal pH and alterations in iso-valeric and valeric acid concentrations, with no measurable changes in total SCFA concentration. The type of FS supplementation had selective effects, with decreased OM digestibility and increased epithelial morphological readout values for the SOY supplement compared with the PALM supplement. Further research will characterize the effect of the type and duration of FS on ruminal epithelial functions, ruminal fermentation, and animal performance with different physiological status, in greater depth.

## Figures and Tables

**Table 1:** Feed ingredients and chemical composition of experimental diets with prilled palm oil (PALM) or soybean oil (SOY) or without fat supplements (CON).

Ingredients, g/kg	Diets ^1^		
	CON	PALM	SOY
Alfalfa hay	200	190	190
Barley grain	660	627	627
Soybean meal	110	104	104
Calcium carbonate	10	9.5	9.5
Sodium bicarbonate	10	9.5	9.5
Vitamin and mineral premix ^2^	5.0	4.7	4.7
Salt	5.0	4.7	4.7
Prilled palm oil ^3^	-	50	-
Soybean oil ^4^	-	-	50
Nutrient composition ^5^			
Dry matter, g/kg	896 ± 20.1	899 ± 18.3	898 ± 18.4
Metabolizable energy (MJ/kg)	11.6	12.5	12.4
Crude protein, g/kg	154 ± 2.1	147 ± 2.1	147 ± 2.2
Ether extract, g/kg	18.9 ± 0.1	67.5 ± 0.1	67.1 ± 0.1
Neutral detergent fiber, g/kg	252 ± 14	239 ± 15	240 ± 14
Acidic detergent fiber, g/kg	135 ± 4.2	127 ± 4.1	127 ± 4.1
Ash, g/kg	67.8 ± 0.31	65.0 ± 0.31	66.5 ± 0.32

^1^ Oils replaced the other dietary ingredients on a proportionate basis. ^2^ The mineral and vitamin premix contained (per kg DM): 500,000 IU vitamin A, 100,000 IU vitamin D, 1 g vitamin E, 180 g Ca, 90 g P, 20 g Mg, 60 g Na, 2 g Mn, 3 g Fe, 0.3 g Cu, 3 g Zn, 0.1 g Co, 0.1 g I, 0.001 g Se and 3 g commercial antioxidant (Globatiox; containing as active ingredients ethoxyquin, propylgallate, and citric acid). ^3^ Commercial palm oil (Energizer RP 10, Malaysia) containing (g/100 g of total fatty acids) C12:0 (0.09), C14:0 (1.26), C15: (0.08), C16:0 (74.4), cis-9 C16:1 (0.06), C18:0 (4.88), *cis*-9 C18:1 (15.1), *cis*-11 C18:1 (0.13), C18:2n-6 (2.82), C18:3n-3 (0.11), and others (1.02). ^4^ Soybean oil containing (g/100 g of total fatty acids) C14:0 (0.1), C16:0 (11.0), cis-9 C16:1 (0.1), C18:0 (4.0), *cis*-9 C18:1 (23.4), C18:2n-6 (53.3), C18:3n-3 (7.18), and others (0.92). ^5^ Analyzed items are means ± SD of three replicates. Metabolizable energy was calculated using a CNCPS-S model (version 1.0.21; Cornell University, Ithaka, NY, USA).

**Table 2:** Dry matter intake (DMI), average daily gain (ADG), feed to gain (F/G) ratio, and carcass weight responses of lambs fed diets with fat supplements (FS; 50 g/kg) of prilled palm oil (PALM), soybean oil (SOY), or without fat supplementation (CON).

Item	Diets			SEM	*p*-Value			
	CON	PALM	SOY		Diet	CON vs. FS	Time	Diet × Time
DMI (g/d)	1513	1721	1702	67.8	0.084	0.028	0.026	0.022
1–7 d	1577	1701	1731	116.1	0.618	0.341		
8–14 d	1625	1904	1834	104.0	0.172	0.071		
15–21 d	1371 ^b^	1706 ^a^	1755 ^a^	101.8	0.032	0.010		
22–32 d	1580	1638	1515	154.5	0.854	0.983		
33–40 d	1390	1672	1757	167.3	0.292	0.130		
41–46 d	1517	1725	1626	168.1	0.687	0.451		
BW (kg)	45.0 ^b^	48.0 ^a^	48.0 ^a^	0.83	0.021	0.006	<0.001	0.008
d 7	42.8	43.0	43.2	0.59	0.916	0.702		
d 14	44.3	45.8	45.8	0.67	0.214	0.083		
d 21	44.5 ^b^	47.0 ^a^	47.6 ^a^	0.60	0.005	0.001		
d 32	45.6	48.8	48.5	0.95	0.056	0.018		
d 40	46.7 ^b^	50.7 ^a^	52.2 ^a^	1.37	0.033	0.012		
d 46	47.0 ^b^	53.0 ^a^	53.5 ^a^	1.26	0.002	0.005		
ADG (g/d)	175 ^b^	312 ^ab^	384 ^a^	20.5	<0.001	<0.001	0.017	0.565
F/G ratio	7.33 ^a^	5.59 ^ab^	4.40 ^b^	0.356	<0.001	<0.001	0.002	0.710
Carcass weight (kg)	23.8 ^b^	25.4 ^a^	26.2 ^a^	0.51	0.012	0.005		
Dressing Percentage (%)	51.2	50.0	50.1	0.75	0.455	0.217		

^a,b^ Values within one row are different if they do not share a common letter *p* < 0.05.

**Table 3:** Apparent digestibility of nutrients in lambs fed diets with fat supplements (FS; 50 g/kg) of prilled palm oil (PALM), soybean oil (SOY), or without fat supplementation (CON).

Item (g/100 g)	Diets			SEM	*p*-Value	
	CON	PALM	SOY		Diet	CON vs. FS
Dry matter	66.7 ^a^	63.6 ^b^	61.4 ^b^	0.43	0.013	0.007
Organic matter	67.6 ^a^	66.9 ^a^	62.04 ^b^	0.56	0.030	0.096
Ether extract	73.3	77.5	78.3	0.66	0.120	0.044
Neutral detergent fiber	64.7 ^a^	61.2 ^b^	59.2 ^b^	0.49	0.023	0.010
Crude protein	74.4	71.5	69.6	0.63	0.155	0.078

^a,b^ Values within one row are different if they do not share a common letter *p* < 0.05.

**Table 4:** Ruminal fermentation characteristics of lambs fed diets with fat supplements (FS; 50 g/kg) of prilled palm oil (PALM), soybean oil (SOY), or without fat supplementation (CON).

Item	Diets			SEM	*p*-Value			
	CON	PALM	SOY		Diet	CON vs. FS	Time	Diet × Time
pH	5.91 ^b^	6.11 ^a^	6.19 ^a^	0.063	0.018	0.007	0.788	0.337
d 13	5.94	6.11	6.18	0.135	0.453	0.235		
d 20	6.11	6.11	6.11	0.128	0.999	0.993		
d 40	5.71 ^b^	6.11 ^a^	6.29 ^a^	0.126	0.011	0.004		
Ammonia-N (mg/dL)	20.4	20.2	24.7	1.44	0.075	0.268	0.019	0.250
d 13	24.6	22.0	24.3	2.35	0.702	0.625		
d 20	18.0	18.9	23.0	1.63	0.096	0.160		
d 40	18.7 ^b^	19.8 ^b^	26.7 ^a^	1.81	0.012	0.054		
Total SCFA (mM)	75.5	77.5	82.2	3.15	0.309	0.267	0.022	0.544
d 13	79.0	71.1	79.8	5.88	0.542	0.612		
d 20	77.2	87.6	91.3	4.98	0.144	0.059		
d 40	70.2	73.6	75.6	5.60	0.791	0.526		
Acetic acid (mol/100 mol)	62.8	63.1	61.4	0.83	0.323	0.579	<0.001	0.079
d 13	62.0	63.9	64.1	0.85	0.187	0.071		
d 20	66.7 ^a^	68.2 ^a^	62.8 ^b^	0.91	0.001	0.291		
d 40	59.8	57.2	57.4	2.09	0.625	0.340		
Propionic acid (mol/100 mol)	21.7	21.3	23.3	1.04	0.388	0.626	<0.001	0.094
d 13	22.8	22.5	19.8	1.54	0.342	0.395		
d 20	18.5 ^b^	16.1 ^b^	21.4 ^a^	1.03	0.007	0.851		
d 40	23.8	25.4	28.7	2.38	0.351	0.273		
Butyric acid (mol/100 mol)	13.6	14.1	13.9	0.96	0.919	0.705	0.259	0.340
d 13	13.3	11.7	14.5	1.21	0.285	0.875		
d 20	13.0	13.6	13.7	0.79	0.799	0.510		
d 40	14.4	17.1	13.6	2.16	0.507	0.725		
Isovaleric acid (mol/100 mol)	0.59 ^a^	0.29 ^b^	0.37 ^b^	0.059	0.005	0.002	0.001	0.013
d 13	0.59	0.34	0.34	0.115	0.236	0.094		
d 20	0.57	0.46	0.70	0.109	0.315	0.916		
d 40	0.63 ^a^	0.078 ^b^	0.08 ^b^	0.057	<0.001	<0.001		
Valeric acid (mol/100 mol)	1.31	1.13	0.99	0.106	0.139	0.075	<0.001	0.007
d 13	1.32	1.58	1.34	0.232	0.694	0.637		
d 20	1.18	1.58	1.39	0.155	0.209	0.121		
d 40	1. 41 ^a^	0.22 ^b^	0.24 ^b^	0.175	0.001	<0.001		

^a,b^ Values within one row are different if they do not share a common letter *p* < 0.05. SCFA, short chain fatty acids.

**Table 5:** Plasma metabolites of lambs fed diets with fat supplements (FS; 50 g/kg) of prilled palm oil (PALM), soybean oil (SOY), or without fat supplementation (CON).

Item	Diets			SEM	*p*-Value			
	CON	PALM	SOY		Diet	CON vs. FS	Time	Diet × Time
Glucose, mg/dL	89.9	87.8	89.8	2.12	0.55	0.55	<0.001	0.18
Cholesterol, mg/dL	153	145	144	5.33	0.44	0.21	<0.001	0.28
Albumin, g/dL	4.10	4.08	4.16	0.18	0.91	0.90	<0.001	0.65
Total protein, g/dL	6.22	6.53	6.31	0.26	0.47	0.38	0.89	0.77

**Table 6:** Eating behavior of lambs fed diets with fat supplements (FS; 50 g/kg) of prilled palm oil (PALM), soybean oil (SOY), or without fat supplementation (CON).

Items	Diets			SEM	*p*-Value	
	CON	PALM	SOY		Diet	CON vs. FS
Eating time, min/d	145 ^b^	208 ^a^	211 ^a^	15.3	0.010	0.003
Eating time, min/kg of DMI	86 ^b^	109 ^a^	116 ^a^	6.88	0.014	0.005
Meal frequency, meals/d	9.0 ^b^	12.1 ^a^	11.4 ^a^	0.78	0.026	0.009
Meal length, min	17.1 ^b^	24.0 ^a^	26.8 ^a^	0.97	<0.001	<0.001
Length of first meal, min	21.4 ^b^	42.1 ^a^	39.2 ^a^	2.67	<0.001	<0.001
Length of second meal, min	17.1 ^b^	20.7 ^b^	32.8 ^a^	3.73	0.020	0.049
Largest meal, min	32.8 ^b^	42.8 ^a^	48.5 ^a^	2.31	<0.001	<0.001
Ruminating time, min/d	361 ^b^	472 ^a^	395 ^b^	20.6	0.004	0.010
Ruminating time, min/kg DMI	229	251	217	15.2	0.306	0.770
Ruminating bouts, bouts/d	15.4	17.0	15.0	0.82	0.223	0.578
Ruminating length, min	31.1	32.0	33.6	2.32	0.756	0.570
Chewing time, min/d	506 ^b^	681 ^a^	606 ^a^	29.7	0.002	0.001

^a,b^ Values within one row are different if they do not share a common letter *p* < 0.05.

**Table 7:** Morphological characteristics of the ruminal papilla of lambs fed diets with fat supplements of (FS; 50 g/kg) prilled palm oil (PALM), soybean oil (SOY), or without fat supplementation (CON).

Items, µm	Diets			SEM	*p*-Value	
	CON	PALM	SOY		Diet	CON vs. FS
Papillary length	2782	3366	3466	218.3	0.082	0.030
Papillary width	667 ^c^	727 ^b^	891 ^a^	35.8	<0.001	0.004
Thickness of the epithelium	233 ^b^	267 ^b^	366 ^a^	20.7	<0.001	0.004
Thickness of submucosal layer	606 ^b^	929 ^a^	975 ^a^	96.9	0.029	0.009
Thickness of the muscle layer	2396 ^b^	3620 ^a^	3616 ^a^	208.5	<0.001	<0.001

^a,b,c^ Values within one row are different if they do not share a common letter *p* < 0.05.

## Data Availability

The data presented in this study are available on request from the corresponding author.
